# Draft genome sequence of cadmium/zinc-resistant and virulent *Staphylococcus aureus* strain BHUSA01 isolated from a wound in Varanasi, India

**DOI:** 10.1128/mra.00862-25

**Published:** 2026-02-03

**Authors:** Shweta Sinha, Sudhir Kumar Singh, Gopal Nath, Durg Vijai Singh

**Affiliations:** 1 Department of Biotechnology, School of Earth, Biological and Environmental Sciences, Central University of South Bihar 206411, Gaya, India; 2 Department of Microbiology, Viral Diagnostic Research Laboratory, Institute of Medical Sciences, Banaras Hindu University 30114, Varanasi, India; University of Pittsburgh School of Medicine, Pittsburgh, Pennsylvania, USA

**Keywords:** *Staphylococcus aureus*, antibiotic resistance, virulence determinants, DNA sequencing, efflux pump

## Abstract

We report the draft genome sequence of *Staphylococcus aureus* strain BHUSA01, isolated from a patient’s wound (pus) at IMS, BHU, Varanasi. The genome is approximately 2.78 Mb in size, has GC content of 32.66%, and coverage of 98.67%, and contains antibiotic resistance, virulence, biofilm-related, and cadmium/zinc resistance genes.

## ANNOUNCEMENT

*Staphylococcus aureus* is a Gram-positive human pathogen frequently implicated in skin and soft tissue infections and is a major contributor to hospital-acquired infections (1, 2). As part of a clinical surveillance study aimed to investigate the genomic basis of *S. aureus* pathogenicity in northern India, we isolated the BHUSA01 strain from a wound (pus) at the Viral Diagnostic Research Laboratory, Institute of Medical Sciences, Banaras Hindu University, Varanasi, India. The strain BHUSA01, identified as *S. aureus,* was isolated from wound (pus) plated on mannitol salt agar and incubated at 37°C for overnight. A single colony was inoculated into Luria-Bertani broth and incubated at 37°C with shaking at 200 rpm for 16 h, and genomic DNA was extracted using a phenol-chloroform-based method described earlier (3).

Whole-genome sequencing was performed using the Illumina NovaSeq 6000 platform, known for high throughput and accuracy (4), which generated 2 × 150-bp paired-end reads, yielding approximately 1 GB of raw sequence data, and using library kit NEBNext Ultra II FS DNA Library Prep Kit (NEB), Index kit: NEBNext Multiplex Oligos (Dual Index Primers Set 1) (NEB). The adapter trimming and quality filtering were performed using FastQC (v0.11.9) (https://github.com/s-andrews/FastQC), based on the total number of Illumina reads obtained from the FASTQC reports of the raw sequencing files (before trimming) (http://www.bioinformatics.babraham.ac.uk/projects/fastqc), having a total of 3,255,083 paired-end reads (R1 = 3,255,083; R2 = 3,255,083) with each read length of 151 bp. The filtered reads were assembled *de novo* with SPAdes (v3.15.5) (https://github.com/ablab/spades/releases/tag/v3.15.5) using default parameters (5). The assembled draft genome was approximately 2.78 Mb in size, had GC content of 32.66%, and coverage of 98.67%, consistent with the genomic characteristics of clinical *S. aureus* isolates reported earlier (6). The total contigs were 186, of which the largest was 434,930, with an *N*_50_ value of 146,965, whereas *N*_90_ was 39667. It was found to be part of clonal complex V and has sequence type 722. Species identity was confirmed by average nucleotide identity (ANI) using FastANI (vl.33) (https://github.com/ParBLiSS/FastANI), showing 98.86% identity with *S. aureus* NCTC8325 with Bio project PRJNA237 and Bio sample SAMN02604235.

**Fig 1:**
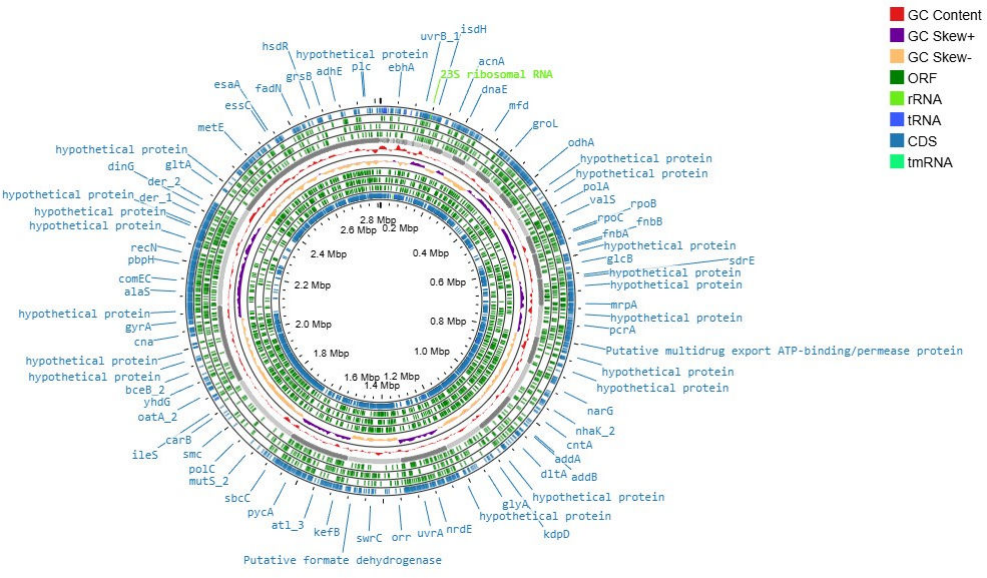
Genomic map for *S. aureus* SABHU01, generated using Proksee (https://proksee.ca/). The map illustrates GC content (red), GC skew (orange/blue), annotated ORFs (light green), CDS (dark green), and RNA features (rRNA in lime green, tRNA in sky blue, tmRNA in cyan).

Annotation was performed using the Rapid Annotations using Subsystems Technology (RAST) server (7), Prokka (v1.14.6) (8), and NCBI Prokaryotic Genome Annotation Pipeline (PGAP v6.5) (9). Figure 1 revealed the presence of 2,571 protein-coding sequences, 58 tRNAs, 71 rRNAs, and several genes associated with key metabolic functions, and *in silico* analysis identified several loci linked to antimicrobial resistance (AMR) and virulence, particularly cadmium/zinc resistance, in addition to putative β-lactamase genes, efflux transporters, and adhesion factors (10, 11). The Resistance Gene Identifier (RGI) (card v6.0) (https://github.com/arpcard/rgi) analysis identified five mechanisms of antibiotic resistance, including antibiotic efflux, inactivation, target alteration, target protection, and target replacement (12). While using the ResFinder 2.5.1 tool (http://genepi.food.dtu.dk/resfinder/job/mb0wm9rk9xo0ilwfxoe4m3t1bd09utzf) from the Center for Genomic Epidemiology, we identified resistance genes associated with multiple AMR classes. Moreover, Virulence Finder 2.0 (https://cge.food.dtu.dk/services/VirulenceFinder/) (Center for Genomic Epidemiology, DTU) identified key virulence determinants, including exoenzymes, hemolysins, enterotoxins, and immune evasion factors, reflecting a high virulence potential of this strain (Table 1).

**TABLE 1:** Identification of virulence determinants, including exoenzymes, hemolysins, enterotoxins, and immune evasion factors, in *S. aureus* strain BHUSA01

Virulence determinant	Presence of gene	Antibiotic resistance
AMR genes	aph(3')-III	Amikacin, iisepamicin, kanamycin, neomycin, lividomycin, paromomycin, ribostamycin, butirosin
	blaZ	Amoxicillin, ampicillin, piperacillin, penicillin
	mecA	Amoxicillin + clavulanic acid, ampicillin + clavulanic acid, cefepime, cefixime, cefotaxime, cefoxitin, ceftazidime, ertapenem, imipenem, meropenem, piperacillin + tazobactam
	dfrG	Trimethoprim
	Msr (A)	Erythromycin, azithromycin, telithromycin, quinupristin
Metal ions	*arsB, arsC*	Arsenic
	*isdH, hssR, hssS, fepC, feuB, feuC, KLKLGEGB 00597, ftnA, irtA, fetB, fetA, sufT, fhuD, salA , yfiZ_1, ctaA, ctaB2, isdB , isdA, isdC, isdE, isdF , isdG_1, frdB , isdG_2 , fur, efeM , scdA, isdI , sodA , yfiY , yfiZ_2, yfhA, mntH_1, sod M*	Ferrous
	*cntA, cntB, cntC, cntD, cntF, nikA, nikB, nikC, nikD, nikE, nixA, ureA, ureB, ureC, ureD1,ureE,ureF, ureG*	Nickel
	*cadC, czcD_1, czcD_2*	Cadmium
	*cntA, cntB, cntC, cntD, cntF, czcD_1, czcD_2*	Cobalt
	*copA, copB, copZ*	Copper
	*mgtE*	Magnesium
	*fosB, mneS, mntA, mntB, mnth_1, mntH_2, mntR, ppaC, scaC, sodA, sodM*	Manganese
	*moaB, mobA, moeA, modB*	Molybedenum
	*aur, cntA, cntB, cntC, cntD, cntF, czcD_1, czcD_2, fosB, ftsH, sufU, yciC_1, yciC_2, yycJ, KLKLGEGB-01297, KLKLGEGB-01687, KLKLGEGB-02076, zntB, znuB, zosA_1, zosA_2, zur*	Zinc
Virulence factor	*aur, hlgA, hlgB, hlgC, lukF-PV, lukS-PV, sea, sec, sec3, seg, sei, sel, sem, sen, seo, seu, scn*	Autolysins, haemolysin, leucocidin, staphylococcal enterotoxins

The presence of cadmium and other antibiotic resistance and virulence genes in this strain highlights the importance of genomic sequencing tools in AMR and virulence gene profiling. The draft genome sequence described here will support not only comparative genomics and evolutionary research but also aid ongoing investigations into AMR and the pathogenicity of *S. aureus* in northern India.

## Data Availability

The draft genome sequence of *Staphylococcus aureus* strain BHUSA01 was deposited to DDBJ/ENA/GenBank under accession number JBOEPY000000000. The raw reads have been deposited in the NCBI SRA under accession number SRR35062318. Additional data can be downloaded from the NCBI BioProject accession number PRJNA1268734.
